# Development of Simple and Affordable Integrating Device for Accurate LED Strip Light Measurement

**DOI:** 10.3390/s25175533

**Published:** 2025-09-05

**Authors:** Krzysztof Skarżyński, Tomasz Krzysztoń

**Affiliations:** 1 Lighting Technology Division, Electrical Power Engineering Institute, Warsaw University of Technology, Koszykowa 75, 00-662 Warsaw, Poland; 2 Kluś Sp. z o.o., Słoneczna 126, 05-506 Kolonia Lesznowola, Poland

**Keywords:** light, lighting, optic, photometry, colorimetry, integrating sphere, goniophotometer, luminous flux, LED strip

## Abstract

**Highlights:**

**What are the main findings?**

- The low-cost integrating device dedicated to LED strips was developed.
- The device offers flexibility in construction and can be built everywhere.

**What is the implication of the main finding?**

- The use of this device is quick and measuring accuracy is sufficient for research and development purposes.
- This device competes with conventional, expensive measurement methods effectively lowering the research and development costs for the LED strip lighting equipment.

**Abstract:**

LED strips are increasingly used as lighting sources in public and private spaces. However, traditional photometric methods, such as integrating spheres, are unsuitable for measuring their light parameters, often resulting in significant errors and requiring expensive instrumentation or calibration. These errors are typically caused by non-uniform illumination of the internal surface or improper internal geometry, especially when measuring LED sources. This article presents the development of a low-cost integrating device specifically designed to measure LED strips’ light parameters. The device is a compact cube with a volume of less than 1.0 m^3^. It was tested against alternative methods using an integrating sphere and a goniophotometer in a professional photometric laboratory. The verification results confirmed its effectiveness. The device showed the maximum relative error of luminous flux measurement to be around 5% compared with the accurate, expensive goniophotometric method. For colorimetric measurements, the maximum Correlated Color Temperature (CCT) absolute error was about 35 K for an LED strip with a CCT of 4000 K, indicating a difference imperceptible to the human eye. These results demonstrate the device’s proper relevance in the research and development of LED strip-based lighting equipment to improve lighting equipment quality and control processes. The device is easy to replicate, significantly reducing production and transportation costs, making it an excellent solution for companies and research units seeking a cost-effective method for LED strip measurements. Additionally, the device can measure other light sources or luminaires with reasonably small sizes emitting light in only one hemisphere. The device is the basis of a patent application.

## 1. Introduction

### 1.1. Luminous Flux Measurement

Luminous flux is a fundamental photometric quantity derived from radiant flux and defined based on the evaluation of this radiation using spectral sensitivity of the human eye adapted to photopic vision (1). This photometric quantity is crucial for the correct design of lighting [1], comparative evaluation of various light sources [2,3], and assessment of energy efficiency in lighting systems [4]. Therefore, ensuring that the luminous flux is measured with the highest possible accuracy for specific lighting applications is essential [5].

(1) $$\phi =K_{m}\int_{0}^{\infty }\phi_{e}\left(\lambda \right)V_{\lambda }\left(\lambda \right)\;d\lambda \;[lm]$$

where

*ϕ*—the luminous flux [lm]
*ϕ*_e_(*λ*)—the spectral distribution of radiant flux [W/nm]
*V*_λ_(*λ*)—the luminous efficiency function for photopic vision [-]
*K*_m_—the maximum luminous efficacy for the photopic vision equals to 683 lm/W
*λ*—the wavelength [nm]

**Figure 1:**
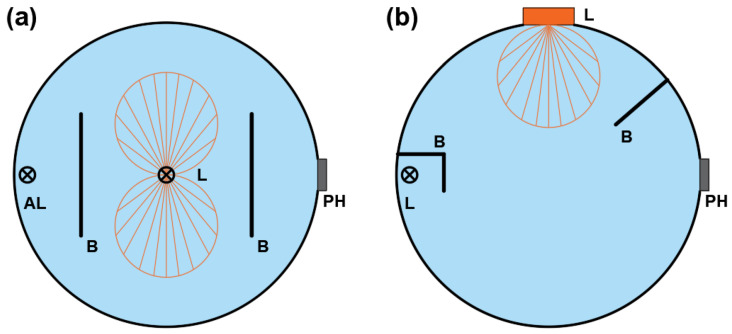
The schemes of integrating spheres with different internal geometries: (**a**) “4π”and (**b**) “2π”; L—tested sample or standard lamp, AL—auxiliary lamp, B—baffle, PH—location of the photometric head.

The primary method for measuring luminous flux involves a comparative measurement of the visible radiation of the test sample with the standard lamp in the integrating sphere [6,7]. It means that the measurement is performed based on indications of the proper head (e.g., photocurrent, illuminance), such as a spectroradiometric probe and knowledge of the actual luminous flux value of the standard lamp. It allows the calculation of the luminous flux value of the test lamp according to Equation (2). Measurement with an auxiliary light source is conducted to eliminate errors related to the different sizes of the sample and standard, which indicates the disturbances in light circulation inside the sphere [8]. Equation (3) considers the factor *i*_ax_/*i*_as_, which is responsible for eliminating this systematic error by using the auxiliary lamp. A typical design of a luminous flux meter is schematically shown in Figure 1a. It is known as the “4π” geometry because it is primarily used for measuring samples emitting luminous flux for almost the entire space [9,10].

(2) $$\phi_{x}=\phi_{s}\frac{i_{x}}{i_{s}}\;[lm]$$

(3) $$\phi_{x}=\phi_{s}\frac{i_{x}}{i_{s}}\frac{i_{as}}{i_{ax}}\;[lm]$$

where:

*ϕ*_x_—the luminous flux of test sample [lm]
*ϕ*_s_—the luminous flux of standard lamp [lm]
*i*_x_—the photocurrent value for test sample [-]
*i*_s_—the photocurrent value for standard lamp [-]
*i*_ax_—the photocurrent value for the test sample illuminated by an auxiliary lamp [-]
*i*_as_—the photocurrent value for the standard sample illuminated by an auxiliary lamp [-]

However, this is not the only possible design solution for an integrating sphere for the luminous flux measurement [11]. The development of various integrating sphere designs has been primarily driven by the advancement of electroluminescent light sources over the past several years [12]. Currently, most single LED applications intended for lighting purposes are made using a surface-mounted device (SMD) or chip-on-board (COB) technology, regardless of whether they are individual chips, modules, or LED strips [13,14,15]. In such applications, the luminous flux is emitted only into one hemisphere. Therefore, a need arose to modify the typical geometry of the luminous flux meter, as the “4π” geometry generates significant measurement errors for such applications. These errors are mainly caused by sphere paint absorption, non-uniform sphere illumination, and unsuitable internal geometry (such as baffle sizes and placement), leading to an increase in errors of up to 10% [16,17]. As a result, “2π” type integrating sphere designs were developed. A typical design of a “2π” geometry luminous flux meter is shown in Figure 1b.

It is important to note that many other dedicated integrating sphere designs exist. These are described in technical standards and may vary slightly depending on the country or region. However, the main ideas of the measurement remain the same:

- The phenomenon of inter-reflections is realized while simultaneously eliminating the direct component of the radiation.
- The internal surface is illuminated with a very high uniformity, which means that the internal surface’s luminance is constant.

Therefore, measuring luminous flux using an integrating sphere seems relatively straightforward. Unfortunately, many factors can disturb a test sample’s luminous flux value. The most critical factors include [18,19]:

- The design of the integrating sphere;
- The sizes and locations of the baffles;
- The type and reliability of standard lamps used;
- The relative position of the standard and the test sample;
- The parameters of the coating applied to the internal surface;
- The size and reflective-transmissive properties of the lamps used;
- The luminous intensity distribution of the standard and the test sample;
- The quality of the photometric head or spectroradiometer (using linearity, spectral, and spatial correction, which are the fundamental photometric requirements);
- Thermal stabilization of the test sample and stabilization of the power supply parameters (which are universal photometric requirements).

All of the above factors influence the price of the integrating sphere. However, the highest costs are related to its size, the coating for the internal surface, and the photometric head or spectroradiometer. According to the authors, the price of a professional “luminous flux meter” (integrating sphere) ranges from several thousand to tens of thousands of dollars. There are also operating costs. Measurements should be checked, and the internal surface should be renewed with adequate coating because it loses its properties during use.

### 1.2. Colorimetric Parameters of Light Sources

The spectral power distribution represents the primary radiometric characteristic of an electric light source. All colorimetric parameters that define the color or the color rendering of the light source are derived from this characteristic [20,21,22,23]. Among the most fundamental colorimetric parameters of light sources are the tristimulus values (x, y) and the correlated color temperature (CCT) [23]. The last measure is required to be included in the technical specification of the lighting product [24].

**Figure 2:**
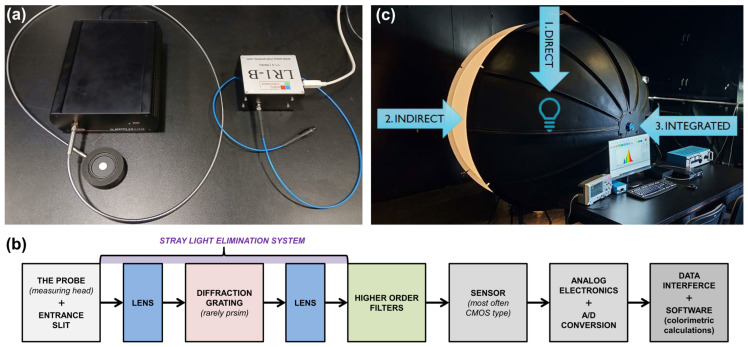
Spectroradiometer: (**a**) exemplary applications, (**b**) typical functional block diagram, and (**c**) schematic concept of light source color measurement setups.

Various spectroradiometers (Figure 2a) measure a light source’s spectrum and its parameters. These devices typically consist of a measuring probe. In this central unit, the optical system, diffraction grating with the appropriate spectral range, higher harmonic filter, measurement sensor (usually CMOS), analog electronics, and software for visualizing measurement results on a computer screen are installed [23,24]. The typical spectroradiometer construction is presented in Figure 2b.

The prices of spectroradiometers range from several hundred to several tens of thousands of dollars, which is related to the quality of the components used and the proper professional calibration for radiation measurements within a given wavelength range. Nevertheless, their various applications have recently dominated the field of lighting measurements, and they are used for many different purposes. In laboratory measurements, spectroradiometers can be used with integrating spheres primarily for measuring luminous flux, thanks to appropriate calibration [25]. However, they are mainly used to study the spectral power distribution from a light source and to determine its typical colorimetric parameters, such as the previously mentioned correlated color temperature.

Measurements can be performed in different configurations, as shown in Figure 2c. It can be performed directly by pointing the probe at the radiation source. However, the measurement may be distorted for some light sources, primarily LED, due to the variable spectrums within the spatial distribution of radiation [26]. Therefore, measurements are taken using the integrating sphere: an indirect method where the probe is directed to the internal surface of the integrating sphere or, ideally, an integrated setup where the probe is mounted in the measurement window of the integrating sphere. The interior of the integrating sphere is coated with a special paint that reflects the radiation in a diffuse and non-selective manner [27]. The difference in the obtained CCT value between the indirect and integrated methods is recommended to not exceed 100 K for the test with illuminant A [16].

### 1.3. Problems with LED Strips and Main Research Aim

It should be emphasized that both integrating sphere designs described in Section 1.1 are unsuitable for measuring highly flexible and bendable applications, such as the currently popular LED strips [28,29]. As mentioned earlier, the professional integrating spheres can be expensive, and their use for measuring the luminous flux of LED strips may result in significant measurement errors with a magnitude of up to 10%. It is mainly because different positions of LED strips, with varying lengths, can disrupt inter-reflections inside the integrating sphere, ultimately affecting the photocurrent values. Goniophotometric systems that allow calculating luminous flux based on the measured luminous intensity distribution are much more appropriate [30], although significantly more expensive. Therefore, the development, implementation, and validation of a new measurement geometry tailored for an integrating device capable of measuring LED strips is necessary. This objective forms the core of the research presented in this article and requires a step-by-step approach involving various laboratory experiments and analyses. The designed device should be compact, simple in construction, cost-effective to produce, and quick and easy to operate. Achieving this goal is feasible, as the literature reports successful attempts at constructing integrating spheres using techniques such as 3D printing [31].

## 2. Materials and Methods

### 2.1. The Idea’s Origin

The idea of creating a simple integrating device dedicated primarily to measuring LED strips arose from collaboration between the Warsaw University of Technology and Kluś. The mentioned lighting company specializes in implementing lighting solutions based on linear luminaires that utilize modern LED strips [31]. The enormous need for rapid, inexpensive, and sufficiently accurate verification of individual samples’ quality in implementing new products led to the creation of a simple, dedicated integrating device.

### 2.2. Planned Research Step by Step

**Figure 3:**
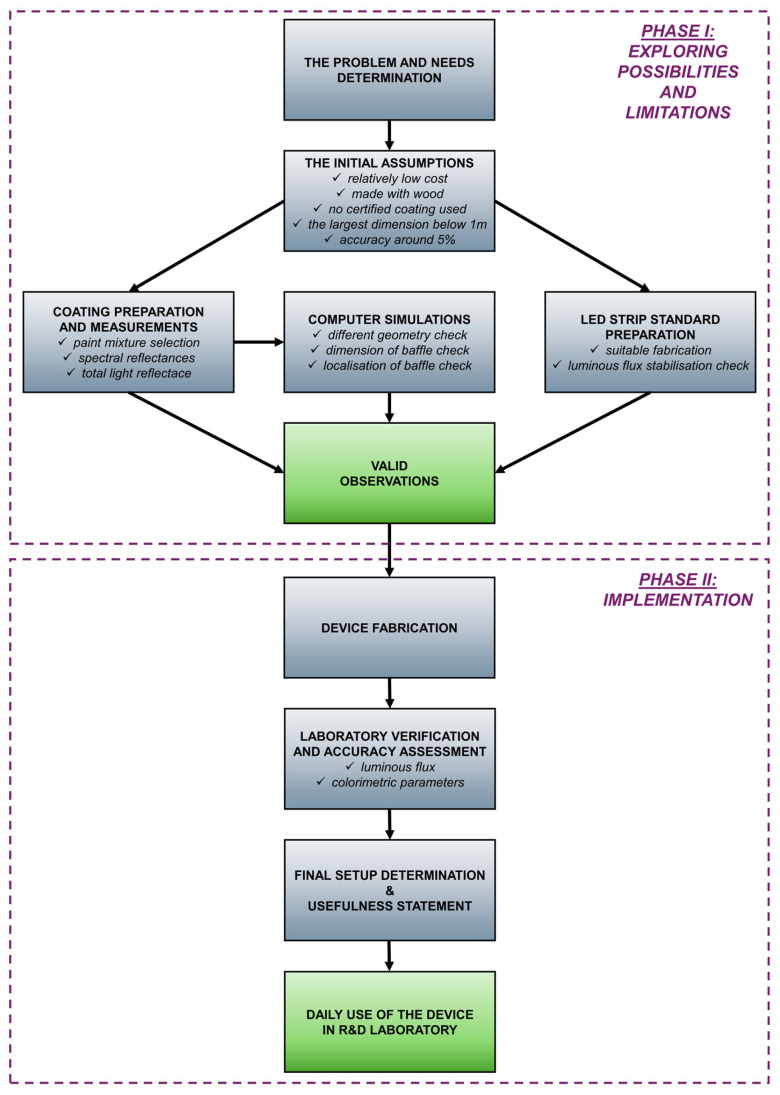
The conducted research step diagram.

The conducted research process was comprehensive. The individual stages of this process are shown in Figure 3. It can generally be divided into searching for possibilities and limitations and implementing the designed device. The problem and need were initially defined: “searching for an alternative, cheap method for measuring the luminous flux of LED strips.” Then, preliminary assumptions were made:

- The device should be inexpensive;
- It should be made of wood or fiberboard (Kluś company has a professional carpentry workshop);
- It should be made without the use of a certified coating for integrating spheres;
- The largest dimension of the device should not exceed 1.0 m;
- The accuracy of luminous flux measurements compared with other laboratories should be around 5.0%.

Next, laboratory measurements of the paint were conducted, and its usefulness was assessed. The process involves a subjective assessment of the paint’s matte finish and objective measurements of the total and spectral reflectance characteristics. A luminous flux standard based on an LED strip. Using LED standards is recommended by the fundamental technical reports such as CIE Technical Report no. 127 [32]. Additionally, the effectiveness of LED standard lams has been confirmed in scientific publications [33]. The luminous flux was tested for stabilization for approximately 200 h, and the operating time was measured using a simple device that recorded the operating hours of the equipment running at the specified rated voltage. Testing the LED strip’s stabilization over 200 h was a compromise, as this duration was arbitrarily chosen. Due to limited testing time, it was impossible to test for at least 10% (6000 h) of the rated lifetime. Moreover, the standard incandescent lamps usually operate for up to about 100 h. Therefore, it was assumed that the prepared standard would be considered sufficiently high-quality if the luminous flux remained stable over 200 h, with no more than a few lumens’ fluctuations. Simultaneously, a computer simulation was prepared to determine whether and how the internal geometry of the device would affect the measurement results. Specially prepared photometric files, described further in Section 3.2, and typical lighting calculation software (DIALux 4.13) were used. The search for possibilities and the limitations phase concluded with the specification of observations and the determination of the internal geometry of the new device.

The prepared device was verified regarding the discrepancies in the results obtained with different positions of the baffle inside its structure and with varying qualities of photometric heads (class L and A). The tests were conducted using two heads of different classes to determine whether the head with poorer spectral correction, available to the company, would produce errors within an acceptable range. This verification was carried out using relative errors (4), with the reference always being the estimated value of the analyzed parameter achieved using different classical methods or devices such as a goniophotometer.

(4) $$\delta =\left|\frac{∆x}{x_{r}}\right|=\left|\frac{x-x_{r}}{x_{r}}\right|\times 100\;[\%]$$

where

*δ*—the relative error [%]
∆*x*—the approximation error [the unit depends on the measure]
*x*_r_—the reference estimated value [the unit depends on the measure]
*x*—the simulated or measured value [the unit depends on the measure]

Comparative measurements were also performed for 12 LED strip samples using the new device and classical measurement methods. These were conducted in a professional photometry laboratory equipped with a calibrated integrating sphere with the primary luminous flux tungsten standard lamp, a spectroradiometer, and a goniophotometer. The laboratory belongs to the Lighting Technology Division of Warsaw University of Technology. This laboratory is not accredited, as it is primarily used for student courses in photometry and colorimetry. However, its staff are specialists in lighting technology and ensure that all measurements are carried out using fundamental principles, good measurement practices, and instruments of appropriate class. The luminous flux and the basic colorimetric parameters (x, y, CCT) were evaluated, and the environmental conditions, such as ambient temperature and humidity, did not affect the results. This time, an analysis was conducted for absolute errors. At the end of the work, specific recommendations were made regarding the applicability of the new device. The device’s overall operational cost in the Kluś laboratory was assessed.

## 3. Results and Discussion

### 3.1. Coating Measurements

The paint used inside integrating spheres is known as “Lambertian coating,” which has the appropriate certification. It must reflect light in an almost entirely diffuse and non-selective manner. Typically, barium sulfate (BaSO4) is used as an additive to white paint during its production [34]. Previous studies [35,36] have reported the successful use of low-cost paint for integrating sphere applications. To reduce the production costs of our device, a custom paint was developed and tested. Commercially available white acrylic water-based wall paint (Dulux Crystal White) was mixed with barium sulfate in weight ratios of 10:1, 8:1, and 6:1. Such small proportions were chosen to ensure that the resulting mixture would not be too brittle. Samples were prepared to test the spectral reflectance distribution by spraying two layers onto bare MDF (medium-density fiberboard), allowing them to air-dry under normal indoor conditions. This application method is the same as that used for the interior surface of the designed device. Note that the technique of paint application can influence reflective properties. So, it was prepared as such to eliminate eventual differences. All prepared samples had a matte finish, as confirmed by subjective organoleptic inspection.

**Figure 4:**
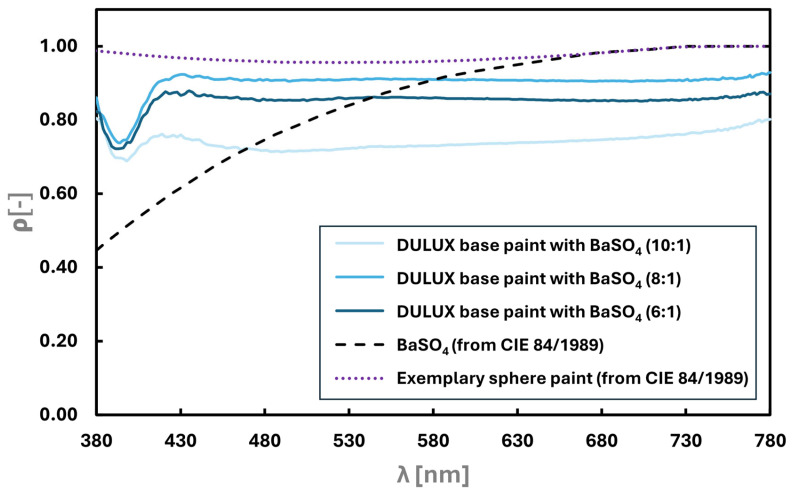
The spectral reflectance distributions of the paint samples, bare BaSO_4_, and exemplary sphere paint retrieved from the CIE 84/1989 technical report [16].

The spectral reflectance distribution was measured using a dedicated reflectometer with a spectroradiometric head, with lighting provided by an incandescent lamp with a color temperature of 2850 K. However, the total reflectance was measured for the LED lamp with a correlated color temperature of 6600 K. This was to eliminate the potential impact of the light source on the measurement results [37] and as the designed device would be used for LED sources only. The characteristics of the obtained spectral distribution of the reflectance coefficient are shown in Figure 4. The values of the total light reflectance coefficients are presented in Table 1.

The measurements indicate that the prepared paint samples have similar properties regarding reflectance selectivity. In each case, the spectral reflectance remains consistent from approximately 420 nm to about 760 nm, but the levels at which stability occurs are different. The ends of the visible spectrum (below 420 nm) showed a significant drop in the spectral reflectance value. However, this could be attributed to typical signal noise issues common in spectroradiometers in these wavelength ranges for the diffraction grating in the visible spectrum or other signal noises [38]. Nevertheless, the characteristics are promising for using the paint in the designed device. The obtained total light reflectance is satisfactory at approximately 0.8 despite the light sources used to illuminate the sample. This level of total reflectance is recommended for integrating spheres [16]. Considering all variables, it was decided to use the 8:1 paint configuration to produce the new device. The 6:1 sample was too brittle and would probably have been too demanding in terms of applying the layer and maintaining the internal walls of the device. In turn, the 10:1 sample had the lowest spectral reflectance levels and was the least stable across the entire spectral range (drop below 420 nm and slight increase above 730 nm).

### 3.2. Computer Simulations

**Figure 5:**
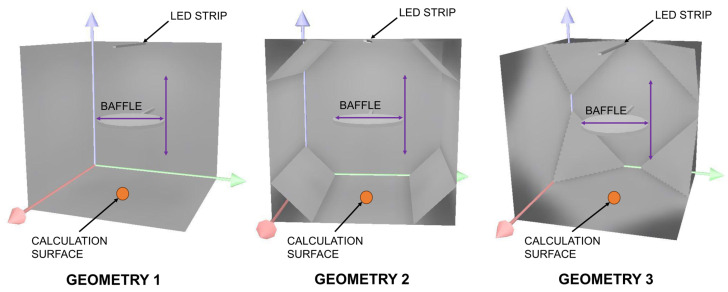
The device’s internal geometries for simulation studies.

By knowing the values of the reflectances, the process of simulating the internal geometry of the designed device was initiated. For this purpose, the DIALux software was employed to design interior lighting. Appropriate models were prepared for three different geometries. Geometry 1 was characterized by only a baffle in the center of the device. Geometry 2 had additionally beveled extended vertical edges of the cube. Geometry 3 had additional beveled corners of the cube. Geometries 2 and 3 resulted from the anticipated need to provide a more uniform illumination of the device’s internal walls. All internal geometries are schematically presented in Figure 5.

**Figure 6:**
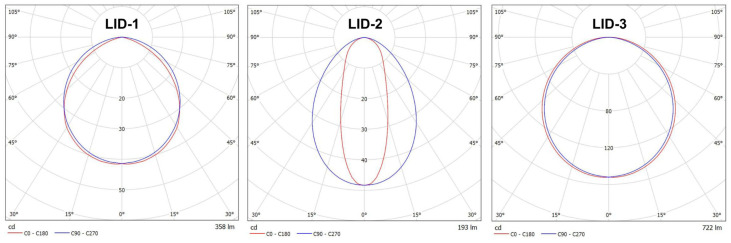
The luminous intensity distribution curves used for simulation studies.

The calculations in the simulation software were performed to simulate the operation of the device in actual conditions according to Equation (2). It is without eliminating the systematic error, as there were exact sizes for the standard and the sample. For this purpose, three different luminous intensity distributions (LIDs) were used, as shown in Figure 6. These distributions had different total luminous flux values: LID-1 had a Lambertian-like distribution (358 lm), LID-2 had a narrower distribution, especially in one plane (193 lm), and LID-3 had an almost Lambertian distribution (722 lm). The reference was the LID of a sample LED strip with a luminous flux of 476 lm and a distribution nearly identical to that of LID-3.

Once all the models and data for the simulation were prepared, a computational process was carried out using variant approach. The variability of the calculations consisted of changing the size of the baffle and its distance from the wall, on which there was a small computational surface representing the measurement window where the photometric head was installed. The sizes of the baffles checked were from a diameter of 20 cm to 40 cm. The distances ranged from 45 cm (approximately half the length of the device) to just 10 cm. Not all baffle sizes were calculated for every distance. The authors’ observations indicated that the smaller the baffle, the closer it had to be to the wall with the photometric head. Otherwise, it would not have effectively eliminated the direct radiation component, which could have dramatically worsened the measurement accuracy.

Additionally, to improve computational accuracy, the luminous intensity distributions were discretized. It means that several photometric files were used to represent the radiation across the entire length of the tested sample. There were precisely 14 photometric files—one for each individual LED chip connected to the half-meter LED strip. It improves simulation accuracy, as using a single photometric file can introduce errors by treating the light source as a single emission point. In reality, each LED emits light from the surface, not a point. This solution is used in professional visualizations and computer lighting calculations [39]. It helps to eliminate certain imperfections associated with the computational algorithms of individual lighting simulation programs [40].

The average illuminance was calculated using nine measurement points behind the baffle (Figure 5). These points were arranged in a polar grid, with one central point and two points on each side. The distance between the outermost points was 5 cm. For every case, the reference values of luminous flux for individual LIDs were used. It made it possible to calculate relative errors and differentiate the obtained configurations regarding predicted measurement accuracy. A summary of the error calculation results is presented in Table 2.

The analysis showed that the proposed internal geometries are relatively similar in terms of the ranges of errors obtained. In each of them, there were configurations where the error was below 0.5%, as well as those where it exceeded 6–7%. It is difficult to determine the exact cause of this. Some limitations of the applied simulation program regarding the computational algorithm implementation, such as not using the energy method, may be responsible. In contrast, more advanced software like Radiance, which utilizes physically based ray tracing and energy methods, would be more suitable for accurate light simulation, especially in the complex internal geometry of the designed device. Nevertheless, the performance of DIALux 4.13 was deemed adequate for preliminary analysis, helping to identify the most promising geometry and configuration before the device’s implementation. The most minor average error was obtained for Geometry 2, with a baffle diameter of 30 cm and a distance from the wall with a head of 25 cm. Moreover, this geometry exhibited high lighting uniformity (above 0.8 for a 5 cm Cartesian grid) on the individual walls. It effectively eliminated the low illuminance levels in the cube’s corners. It is further promising in appropriately shaping inter-reflections in the designed device. Furthermore, Geometry 2 is very easy to construct. Therefore, it was decided to build the device based on this internal geometry, with the condition that the impact of baffle size and location would be experimentally verified in the implemented device.

### 3.3. LED Strip Standard Preparation

Using an appropriate luminous flux standard is essential for performing luminous flux measurements correctly, ensuring the required measurement reliability. In classical integrating spheres, light sources are typically incandescent tungsten lamps. However, there is an increasing consideration of LED light sources being standard lamps [33].

**Figure 7:**
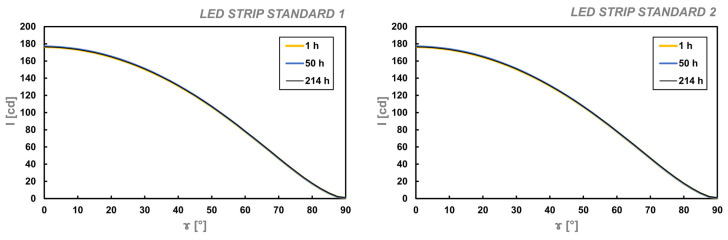
The average luminous intensity distribution curves of LED strip standard for different operation times. Remark: The obtained curved are of the same shape for each time and samples which proves the high accuracy of the goniophotometric system used.

In the designed device, using a tungsten lamp would not be reasonable. Naturally, the idea of creating a luminous flux standard based on an LED strip emerged. For this purpose, a strip of LED was selected from the Kluś company’s offer, which exhibited the best quality. It means that customers did not return this LED strip due to damage, did not report any color changes, and its rated durability was 60,000 h (see Appendix A.1). Two samples of this LED strip, each 50 cm long, were prepared. These were subjected to short-term stabilization testing of luminous flux over time during operation, meaning they were operated for over 200 h, powered by 24 VDC (Korad KKG305D with a voltage and current resolution of 0.01 and 0.001, respectively). For three different time points (1 h, 50 h, and 214 h), the luminous intensity curves were measured for each, using a high-quality SpectroColor goniophotometer with an angular resolution of 0.01 degrees and a photometric head of class A. The measurement distance was 6.0 m, and the operation condition was normal. The selected time slots determined the laboratory’s operating hours and space availability between scheduled teaching sessions. The luminous flux was then calculated. The results of these measurements and calculations are presented in Figure 7 and Table 3.

The selected samples for the luminous flux reference based on the LED strip meet the desirable stability over time. The results obtained demonstrate stability over time. Across different time points, the current fluctuation did not exceed 3 mA (1.56%), whereas the luminous flux was within 3 lumens (0.57%), the generally accepted level for short-term stability. These discrepancies are minimal and can be considered negligible. Therefore, the references prepared in this manner are sufficiently reliable and can be used as a standard for basic calibration of the designed device. It means using the luminous flux calculation in Equation (2), and it is possible because the LED strip standard and sample dimensions are almost identical.

### 3.4. Specific Device Configuration

**Figure 8:**
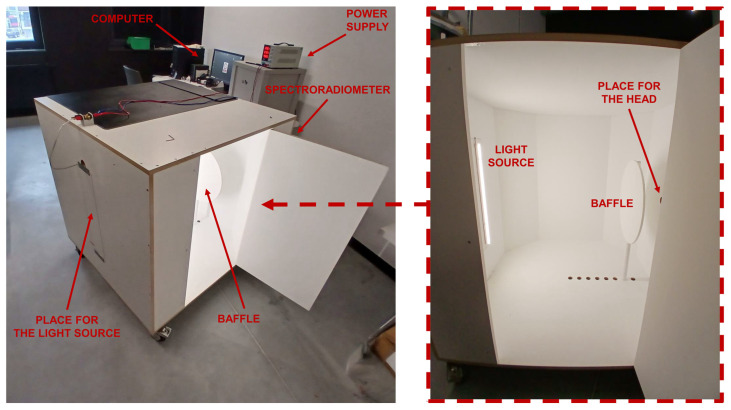
The final appearance of the device.

All the work described in the previous subsections was used to create a simple integrating device dedicated to measuring LED strips. The appearance of the device and the entire measurement setup are shown in Figure 8.

Experimental verification was conducted to verify its functionality and determine the optimal position of the baffle inside the device. It was carried out for a baffle with a diameter of 30 cm and two different photometric heads. The first was class L, meaning its spectral match error was below 1.5%. The second head was class A, with a spectral match error of less than 3%. Additionally, it allowed for the measurement of colorimetric parameters–chromaticity coordinates x, y, and CCT. By measuring the luminous flux of an LED strip with known photometric and colorimetric parameters, the relative error was determined for various baffle positions, ranging from 10 cm to 45 cm from the wall with the photometric head, with a step of 5 cm. The measurement results obtained are presented in Table 4.

The most significant relative error in the luminous flux measurement, around 3–4%, was observed for the longest distance between the diaphragm and the wall with the measurement head. On the other hand, minor errors were obtained for the closest baffle positions, 10 cm and 15 cm, using the class L head. The 20 cm and 25 cm distance errors were also acceptable for the class A head, as they were only around 1.0%. In general, slightly higher error values were obtained than those predicted during the computer simulation phase, which is entirely understandable due to limitations of lighting simulations.

In the case of colorimetric parameter measurements, the highest relative errors appeared for baffle positions of 10 cm, 20 cm, and 25 cm. However, these errors are relatively small and do not exceed 2.3%. It can be considered a satisfactory result. The most negligible relative errors for the chromaticity coordinates x, y, and CCT, respectively, 0.79%, 1.83%, and 0.14%, were obtained for a distance of 15 cm. The baffle position at 15 cm from the wall with the photometric head is characterized by the slightest error values for all analyzed photometric and colorimetric parameters. For this reason, it was selected as the most appropriate position, and the device operated sufficiently in this configuration.

### 3.5. Verifying Luminous Flux and Color Measurements

To accurately verify the functionality of the designed integrating device dedicated to LED strips, it was decided to conduct verification on 12 different LED strip samples (for more technical details, please look at Appendix A.2). The samples varied in terms of their light parameters. The lowest luminous flux value of the verification samples was 325 lm, and the highest was 1150 lm. The verification samples also had four different CCT values: 2700 K, 3000 K, 3500 K, and 4000 K, meaning they had different spectral distributions. The set of samples adequately represented the range of these parameters for typical 50 cm LED strip lengths. Table 5 presents the sample lists, and more details are included in Appendix A.2.

All samples were measured using the designed device, a classical integrating sphere, and the goniophotometric method in a professional photometric laboratory. The absolute error values were then calculated, meaning the differences between the values obtained with the designed device and those measured in another laboratory using dedicated instruments and measurement methods (Table 5).

**Figure 9:**
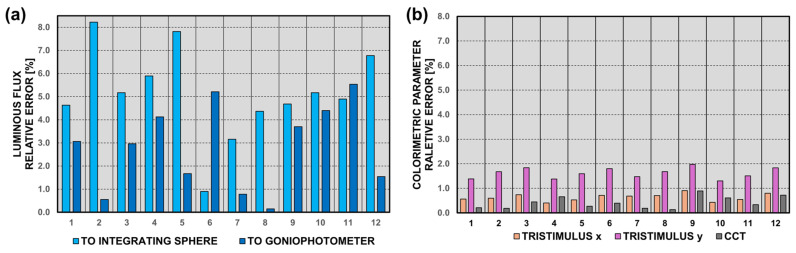
The obtained relative errors during the verification tests in the photometric laboratory: (**a**) for luminous flux measurement using integrating sphere and goniophotometer, and (**b**) for colorimetric measures by the spectroradiometer with the integrating sphere.

The minimum absolute error in the luminous flux measurement compared with the integrating sphere measurements is 10 lm, while the maximum is 83 lm. Compared with the goniophotometric method, these errors decrease and range from 2 lm to 57 lm. The relative errors for the integrating sphere reference exceed 8%, while for the goniophotometric method, they average 5.5% (Figure 9). These error values are acceptable, especially considering other factors influencing the luminous flux measurements, such as the power supply fluctuations, LED operating temperature, or other environmental conditions.

In the case of verifying the accuracy of colorimetric parameter measurements, the results from the designed device were compared only with measurements taken with an integrating sphere painted inside with certified laboratory paint. It should be emphasized that in both cases, measurements were performed using the same model of spectroradiometer (GL Optic Spectis Touch 1.0 + Flicker). The absolute errors obtained regarding the chromaticity coordinates, which were below 0.008, were very satisfactory. This is because they are at a similar level to the errors specified in the technical datasheets of professional spectroradiometers. It depends on what the measurements made with the new device will be used for. In the case of advanced colorimetric analysis, color space shaping, or the analysis of color rendering parameters, the accuracy may be insufficient. However, for creating product catalog sheets used by engineers and lighting designers, the measurement accuracy of basic colorimetric parameters is excellent. This is further supported by the fact that the maximum absolute error for the CCT was 35 K for a 4000 K LED strip. The human eye does not notice such a slight color difference at such a high CCT level [41]. The obtained relative error values are shown in Figure 9. The errors for all verification samples are below 2.0%, and for the CCT, they are even below 1.0%.

The verification tests performed on the actual device using LED strip samples proved the correctness of the designed device’s operation. Its usefulness in the R&D process of lighting products with LED strips as a light source and increasing the credibility of lighting system designs using LED strips is also very high.

### 3.6. Simplified Cost Analysis and R&D Suitability

An essential aspect of the presented device for measuring the light parameters of LED strips is its cost. The price of a typical integrating sphere, depending on its size, configuration, and equipment, including transport costs, was at least USD 6000 in 2017 [42]. Currently, prices in Europe are even higher. Professional setups for measuring luminous flux with an integrating sphere with a diameter of 1.0m, a spectroradiometer, and proper calibration (or a set of appropriate reference lamps) cost around USD 20,000. Goniophotometric systems are even more expensive, starting at approximately USD 30,000.

In contrast, the cost of producing the designed device discussed in this article was only about USD 500 (without auxiliary instrumentation). It is because its geometry was made from MDF (medium-density fiberboard), and the interior was coated with commercial paint that had previously been tested for suitability in photometric measurements. Additionally, the device requires a laboratory power supply for the tested samples, which costs about USD 100 if using a DC transformer power supply. A good class A photometric head or an average-quality spectroradiometer (USD 1000) should also be considered. All of this brings the device’s total cost to about USD 1600. This is about 12 times less than conventional, professionally calibrated measurement setups, which usually do not have the proper geometry, as they are typically 4π type integrating spheres. The costs are minimal if a lux meter or spectroradiometer is already available. However, it must be emphasized that the total cost strongly depends on the auxiliary instrumentation. The presented expenses are average values, and if a more expensive photometric head or power supply is used, the total cost will increase proportionally. Using a good quality, stable DC power supply is particularly recommended to eliminate as many ripples as possible in the electrical parameters powering the probe and the test sample. Similarly, having the best possible photometric head is desirable, primarily regarding spectral mismatch correction. It is important to note that this cost analysis does not consider all the tiny factors (the cost of paint and barium sulfate) or those that are impossible to estimate (the labor involved in painting and assembling the device). These factors are negligible in R&D or academic environments, where technicians are responsible for this. However, in reality, the costs of manufacturing the device may be slightly higher, but they will always be significantly lower than purchasing a calibrated integrating sphere or goniophotometer system. For instance, the Kluś company recouped the device in just 2 months after its implementation, and the high measurement stability. Additional benefits included speeding up the measurement process and eliminating the hassle of packaging and shipping samples to another research laboratory.

## 4. Conclusions

The article discusses issues related to measuring the luminous flux of LED strips. It presents the development, design, construction, and verification of a new, simple integrating device dedicated to such light sources. The device allows for measuring samples up to 50 cm in length under normal room conditions (ambient temperature 25 °C, humidity about 80%), after a stabilization period that usually lasts up to 30 min. It can be an effective alternative to professional photometric instruments such as integrating spheres and goniophotometric systems. Its accuracy is approximately 5% compared with measurements obtained in a photometric laboratory using standard instruments, which falls well within the generally accepted error margin of 10% for luminous flux measurements and has been achieved in various applications [17]. All the work resulted in creating an easy-to-use device successfully used by one lighting company’s research and development department. The presented research and analysis results prove that integrating a device with a shape other than a sphere is possible, achieving sufficiently high measurement accuracy. A specific limitation of this device is the need for a photometric or spectroradiometric head, which further increases its costs. However, these are now widely available worldwide, and their costs are not prohibitive. On the one hand, using a measurement head, even of low quality, will result in more significant measurement errors and lower confidence in the results’ absolute values.

On the other hand, a person with appropriate knowledge or experience in photometric research will be able to differentiate the results and identify samples with the best quality. It is also worth noting that the proposed solution is simple to replicate, which means it can be built almost anywhere worldwide, reducing transportation costs. It is essential to pay attention to the proper selection of the internal frame, the uniformity of lighting inside, and the location of the baffle. Alternative device variants are the basis for further research related, for example, to significant miniaturization of the device or its fabrication using 3D printing technology and light sensors commonly available in the electronic component market. It could further reduce costs, removing the barriers to using photometric measurement equipment, which would undoubtedly improve the quality of the lighting equipment used, primarily LED chips, modules, and strips.

Additionally, other device geometries, locations, and sizes of internal apertures could be investigated, as well as attempts to implement an auxiliary light source or find a better coating. It would also be beneficial to systematize the generation of luminous flux patterns based on LED strips. Additionally, calculating the total measurement uncertainty budget would further improve the accuracy of measurements performed using this device.

## 5. Patents

The solution described is the subject of a patent application submitted to the Polish Patent Office on 3 March 2024, under *No. P.448746: “Device for measuring the luminous flux of a LED strip and LED profile”* by the authors of this paper.

## Figures and Tables

**Table 1:** The measurement results of the total light reflectance for the prepared samples illuminated by different light sources (values in %).

Lamp Type	Paint Sample		
	1:10	1:8	1:6
Incandescent lamp—2850 K	82.8	81.6	79.7
LED lamp—6600 K	82.0	82.0	77.2

**Table 2:** The summary of relative errors obtained in simulations of the influence of the size and position of the baffle inside the designed device (values in %). The red color represents the largest relative errors, while the green color is the smallest.

Lid Type	Baffle Size [cm]/Distance from the Wall [cm]													
	40/45	40/40	40/35	40/30	40/25	40/20	40/15	40/10	30/25	30/30	30/15	30/10	20/15	20/10
**Geometry 1**														
LID-1	0.24	0.08	0.55	0.64	1.23	1.47	2.18	2.22	0.53	1.01	1.41	2.54	0.89	1.68
LID-2	2.29	2.05	1.46	0.40	0.59	2.50	5.40	6.87	0.56	2.33	5.17	9.07	4.05	9.42
LID-3	0.40	0.39	0.24	0.16	0.25	0.43	0.28	0.67	0.08	0.39	0.58	0.47	0.15	0.40
AVG	0.98	0.84	0.75	0.40	0.69	1.47	2.62	3.25	0.39	1.24	2.39	4.03	1.70	3.83
**Geometry 2**														
LID-1	0.85	0.85	0.64	0.14	0.22	0.16	0.08	0.89	0.55	0.35	0.15	1.43	0.61	0.57
LID-2	2.51	2.81	2.08	1.24	0.37	1.52	3.65	6.85	0.44	1.25	3.96	8.10	2.64	7.37
LID-3	0.43	0.14	0.07	0.29	0.52	0.78	0.93	0.49	0.27	0.35	0.90	0.59	0.47	0.57
AVG	1.26	1.27	0.93	0.56	0.37	0.82	1.55	2.74	0.42	0.65	1.67	3.37	1.24	2.84
**Geometry 3**														
LID-1	1.45	1.82	0.46	0.20	0.41	0.95	1.11	2.14	0.13	0.40	0.77	2.44	0.06	1.21
LID-2	4.95	5.59	3.79	3.73	2.27	0.16	1.69	4.42	2.67	1.05	1.77	6.12	0.24	4.84
LID-3	0.26	1.04	0.46	1.27	0.83	0.66	0.79	0.46	0.64	0.79	0.77	0.24	0.57	0.67
AVG	2.22	2.82	1.57	1.73	1.17	0.59	1.20	2.34	1.15	0.75	1.10	2.93	0.29	2.24

**Table 3:** The luminous fluxes of the prepared LED strip standard for different operation times.

t [h]	LED Strip Standard—Sample 1			LED Strip Standard—Sample 2		
	$U_{N}\;[V]$	$I_{N}\;[mA]$	ϕ [lm]	$U_{N}\;[V]$	$I_{N}\;[mA]$	ϕ [lm]
1	24.05	192	522	24.05	193	522
50	24.08	192	524	24.08	193	523
214	24.05	195	525	24.05	195	525

**Table 4:** Results of the impact of baffle size, location, and photometric head quality on luminous flux measurements in the created device (values in %). The red color represents the largest relative errors, while the green color is the smallest.

Relative Error	Photometer		Distance from the Wall [cm]							
	Class	$f_{1}\prime \;[\%]$	10	15	20	25	30	35	40	45
$\delta_{\phi }\;[\%]$	L	<1.5	0.52	0.26	1.87	1.17	0.66	0.61	0.39	4.11
	A	<3	1.52	1.34	1.03	1.08	2.32	1.19	2.24	3.01
$\delta_{x}\;[\%]$			2.04	0.79	1.77	1.70	1.65	1.63	1.61	1.61
$\delta_{y}\;[\%]$			2.21	1.83	1.98	1.96	1.93	1.93	1.93	1.91
$\delta_{CCT}\;[\%]$			2.40	0.14	2.02	1.89	1.82	1.72	1.72	1.72

**Table 5:** Results of the absolute errors for different photometric and colorimetric measures obtained during the laboratory verification tests. The red color represents the largest absolute errors while the green color is the smallest.

LED Strip	1	2	3	4	5	6	7	8	9	10	11	12
**Basic Data of LED Strips for Verification Tests**												
CCT [K]	3000	3500	4000	3000	3500	4000	2700	3000	4000	2700	3000	4000
ϕ [lm]	325	330	348	963	978	1020	360	383	400	1115	1138	1150
**Integrating Sphere**												
∆ϕ [lm]	16	33	21	60	83	10	11	16	16	51	49	71
∆x [-]	0.0025	0.0025	0.0029	0.0018	0.0022	0.0028	0.0032	0.0031	0.0035	0.0020	0.0024	0.0031
∆y [-]	0.0056	0.0066	0.0071	0.0056	0.0063	0.0070	0.0061	0.0068	0.0076	0.0054	0.0061	0.0071
∆CCT [K]	6	6	17	19	9	15	5	4	35	16	10	28
**Goniophotometer**												
∆ϕ [lm]	11	2	12	42	18	57	3	1	13	44	55	16

**Figure A1:**
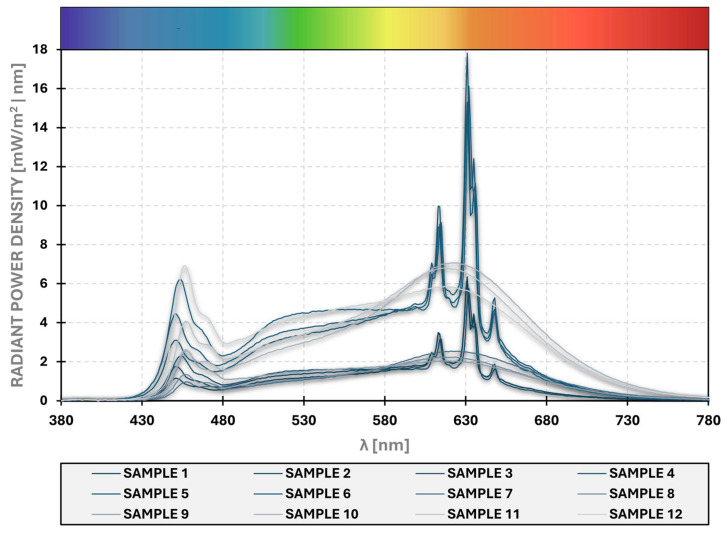
The spectral power distribution for 12 test samples.

## Data Availability

Data will be made available on request.

## Appendix A

### Appendix A.1. LED Strip Technical Specification

The technical specification of the LED strip selected can be found in European Product Registry for Energy Labelling (EPREL) with the following link: https://eprel.ec.europa.eu/screen/product/lightsources/689701: Retrieved on 19 August 2025.

### Appendix A.2. Additional Colorimetric Data for 12 Test Samples of LED Strips

**Table A1:** The values of colorimetric parameter (tristimulus values x, y, correlated color temperature CCT, distance from the black body locus Duv, and color rendering index CRI) for the 12 test samples of LED strips.

Sample	x [-]	y [-]	CCT [K]	Duv [-]	CRI
1	0.4424	0.3995	2872	0.002549	94.6
2	0.4156	0.3874	3254	0.003578	95.5
3	0.3886	0.3795	3806	0.001054	95.9
4	0.4431	0.4010	2872	0.002042	95.0
5	0.4138	0.3894	3308	0.002369	96.2
6	0.3897	0.3815	3793	0.000407	96.8
7	0.4647	0.408.	2613	0.001355	92.6
8	0.4360	0.3984	2969	0.002197	92.3
9	0.3818	0.3770	3965	0.000319	93.3
10	0.4660	0.4095	2608	0.000895	92.5
11	0.4366	0.3982	2957	0.002345	91.9
12	0.3844	0.3802	3920	0.000447	93.8
